# Taxonomic revision of *Habenaria josephi* group (sect. Diphyllae s.l.) in the Pan-Himalaya

**DOI:** 10.3897/phytokeys.175.59849

**Published:** 2021-04-06

**Authors:** Tirtha Raj Pandey, Xiao-Hua Jin

**Affiliations:** 1 State Key Laboratory of Systematic and Evolutionary Botany, Institute of Botany, Chinese Academy of Sciences, Beijing 100093, China Institute of Botany, Chinese Academy of Sciences Beijing China; 2 University of Chinese Academy of Sciences, Beijing 100864, China University of Chinese Academy of Sciences Beijing China; 3 National Herbarium and Plant Laboratories, Godawari, Lalitpur, Nepal National Herbarium and Plant Laboratories Lalitpur Nepal

**Keywords:** Distribution, lectotypes, morphological characters, neotype, taxonomy

## Abstract

Species of the *Habenaria josephi* group in the Pan-Himalaya region are revised, based on their morphological characters and results of previous molecular phylogenetics. Eight distinctive species are recognised; key to the species, taxonomic descriptions, illustrations and distribution maps are provided. *Habenaria josephi* is re-instated, based on morphological and molecular evidence; *H. wolongensis* is synonymised with *H. aitchisonii*, a neotype for *H. tibetica* and the lectotypes for *H. balfouriana*, *H. fargesii*, *H. glaucifolia* and *H. clarkei* are designated.

## Introduction

*Habenaria* Willd. is a large genus in the Orchidaceae (Orchidoideae, Orchideae, Orchidinae), with about 891 species (Govaerts et al. 2020), most of which are terrestrial plants. The genus is distributed along the tropical, subtropical and temperate zones of the Old and New Worlds (Pridgeon et al. 2001; Batista et al. 2011) and has three main centres of diversity, i.e. eastern Asia, central and southern Africa and Brazil (Kurzweil and Weber 1992). The plants are characterised by frequently having simple or bifid petals, a tripartite lip, long rostellar arms, stalked stigmas and a well-developed nectariferous spur (Dressler 1993; Pridgeon et al. 2001).

Of about 208 species of *Habenaria* occurring in the south to east Asian biodiversity hotspot, roughly one fifth are represented in the Pan-Himalaya (Pearce and Cribb 2002; Chen and Cribb 2009; Rajbhandari and Rai 2017; Govaerts et al. 2020). Amongst the Asian clades of Old World *Habenaria*, one group with two basal leaves and with a temperate or alpine distribution is of particular taxonomic interest: the *Habenaria josephi* Rchb.f. complex, as the species belonging to this group formed a close alliance (clade XXIV) in a recent molecular study (Jin et al. 2017). Additionally, they share many morphological traits (e.g. pubescent scape and floral parts, mostly 2-lobed petals) and similar habitats, which often renders the species delimitation difficult; owing to this, some species were either misidentified or assigned different ranks in the past (Lang 1984; Pearce and Cribb 2002; Lucksom 2007; Chen and Cribb 2009; Choudhury et al. 2011; Maity et al. 2019). To address these taxonomic inconsistencies, here we attempt to revise the group in light of the recent molecular works (Jin et al. 2017; Raskoti and Ale 2019) and a broader examination of the herbarium specimens and literature. Our study shows eight species from the group occurring in the region, for which general morphological features, taxonomic description and illustrations are provided. Furthermore, a brief history of Habenaria section Diphyllae Kränzl. and an artificial key to the species are also given.

## Materials and methods

### Study area

The study area Pan-Himalaya (also referred to as the PH hereafter) ranges from parts of Afghanistan in the west to the Yunnan Province of China in the east, forming a natural phytogeographic unit; it is further divided into 17 subregions (Hong 2015, Fig. 1).

**Figure 1.**
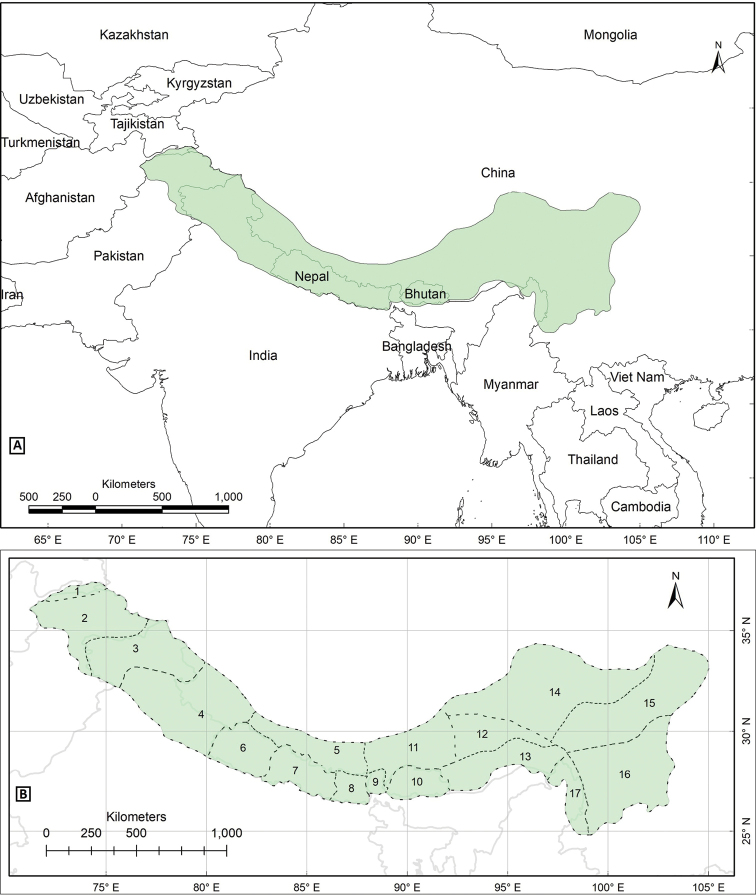
Biogeographical context of the study, the Pan-Himalaya **A** geographic location of the Pan-Himalaya (shaded with green colour) **B** subregions of the Pan-Himalaya (Hong 2015) **1** Vakhan **2** N Pakistan **3** Jammu and Kashmir **4** U Ganga and Indus **5** U Yarlung Zangbo **6** W Nepal **7** C Nepal **8** E Nepal **9** Sikkim and Darjiling **10** Bhutan **11** M Yarlung Zangbo **12** L Yarlung Zangbo **13** Yarlung Zangbo-Brahmaputra **14** Tangut **15** N Hengduan **16** S Hengduan **17** U Irrawaddy.

This work is based on the review of relevant literature and examination of herbarium specimens, supplemented with observations made on living plants in natural habitats. The specimens of *Habenaria* collected from the PH (Fig. 1), preserved at the herbaria AMES, B, CAL, E, K, KATH, KUN, LD, LE, P, PE, S, TI, TUCH, UPS, W and WU (herbarium acronyms according to Thiers 2020) were thoroughly examined; CAL, KATH, PE and TUCH were personally visited and for others, online catalogues were utilised (e.g. ‘www.cvh.ac.cn’ for Chinese herbaria) and high-resolution images of putative type materials were requested.

More than 500 specimens were sorted and about 200 of them, occurring within the PH, were considered for taxonomic characterisation. Species descriptions are based on vegetative and reproductive features as observed directly and/or through stereomicroscopes (Nikon SMZ1000 and Leica S8 APO) for details. Floral parts were rehydrated in boiling water before their observation and measurements were made under the microscopes. Lip and spur morphology was particularly regarded as taxonomically-informative characters. For species delimitation, morphological species concept (Cronquist 1978; Stuessy 2009), along with recent molecular phylogeny (Jin et al. 2017), was taken into consideration.

Data on phenology, habitat and distribution were derived from specimen labels and the distribution maps were prepared from the occurrence locations approximated to the corresponding PH county or district; the list of examined specimens is arranged in the geographical order of the PH (Hong 2015). Information on distribution outside the PH and on illustrations available from literature are also provided.

## Results

### The taxonomic history of Habenaria sect. Diphyllae

Habenaria sect. Diphyllae is one of the 32 sections established by Kränzlin (1892) in his first worldwide revision of *Habenaria*, including 17 species from Africa and Asia. The sections he assigned were based on the degree of dissection of the petals and labellum and on the structure of the gynostemium. The presence of 3-lobed labellum, simple or bilobed broad petals and thick fleshy stigmatic processes were taken as the diagnostic characters for the section Diphyllae (Kränzlin 1892; Kränzlin 1901). Later, Summerhayes (1968) designated *H. diphylla* as the type for the section and ascribed 24 species from East Africa to it. Several sectional treatments of *Habenaria* are available for Neotropics and Africa (Batista et al. 2013; Summerhayes 1968), but comprehensive accounts of Asian species are lacking, though country-level treatments were attempted previously (e.g. Pearce and Cribb 2002). The present study has uncovered the occurrence of 11 species of H. sect. Diphyllae in the PH. Eight of these species are found in high mountain habitats (i.e. collected at an altitude above 1500 m) and represent a monophyletic clade, which is here designated as the *H. josephi* group. The three remaining species (*H. reniformis*, *H. diphylla* and *H. acianthoides*), in contrast, inhabit the tropical climate and are morphologically distinct (e.g. glabrous scape and floral parts, petals always simple) and will not be considered here.

### General morphology

**Figure 2.**
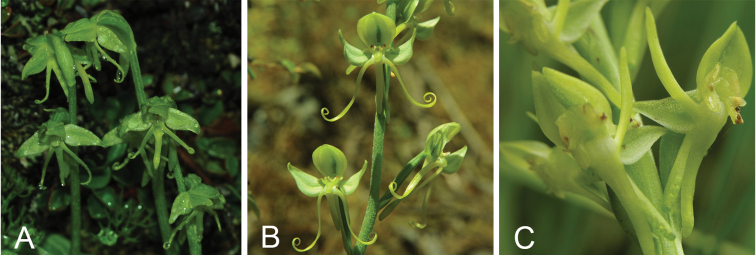
Floral structure in *Habenaria josephi* group **A***H. josephi***B***H. glaucifolia***C***H. aitchisonii* (Photographs by X.H. Jin).

**Tuber**: One or two, globose, oval or elliptic, underground, with few roots dispersed around their junction to the stem. **Stem**: Erect, terete and slender (sometimes robust in *Habenaria aitchisonii*), pubescent, often papillate in *H. diplonema*, *H. aitchisonii*, *H. szechuanica* and *H. tibetica*. **Leaves**: Typically two, opposite to sub-opposite, appressed to the ground, glabrous (densely papillate hairy in *H. diplonema*). Leaf blades orbicular or ovate-orbicular, with amplexicaul base and acute or mucronate apex (acuminate in *H. diplonema*). **Inflorescence**: Racemose, with few (two to six in *H. josephi*; up to eight in *H. glaucifolia*, *H. fargesii* and *H. szechuanica*; up to 14 in *H. diplonema*, *H. balfouriana* and *H. tibetica*) to many-flowered peduncle (sometimes reaching up to 40 flowers in *H. aitchisonii*). Floral bracts are mostly lanceolate with acuminate apex and densely pubescent. **Ovary and pedicel**: Pubescent, sometimes papillate (e.g. *H. aitchisonii*, *H. balfouriana*, *H. szechuanica* and *H. tibetica*); curved and twisted. **Flower**: Generally small-sized (smallest in *H. diplonema*, larger in *H. glaucifolia*, *H. szechuanica* and *H. tibetica*), greenish to greenish-yellow (Fig. 2), sometimes fragrant (e.g. *H. diplonema* and *H. josephi*). Dorsal sepal forms the hood together with the petals, while lateral sepals are deflexed. Six of the species are with distinct 2-lobed petal; *H. fargesii* has filiform, long anterior lobe; in *H. glaucifolia*, anterior lobe is lanceolate and smaller than posterior lobe and in *H. aitchisonii*, *H. balfouriana*, *H. szechuanica* and *H. tibetica*, it is represented by a small tooth at the base of the petals. *H. diplonema* and *H. josephi* are with entire petals, albeit the basal part is broadened. Lip shape is a taxonomically-informative feature in this group; though all the species possess 3-lobed lips, the relative size and orientation of especially the lateral lobe is quite variable; *H. glaucifolia*, *H. szechuanica* and *H. tibetica* have much longer lateral lobes which ultimately are coiled at the tip; lateral lobes of *H. aitchisonii* and *H. balfouriana* are reflexed up, while those in *H. josephi*, *H. diplonema* and *H. fargesii* are deflexed. A prominent needle-like appendage present at the base of the lip near the opening of the spur is characteristic of *H. szechuanica*. Spur, too, exhibits remarkable variation; *H. diplonema* bears a very short spur, in *H. aitchisonii*, spur is shorter than ovary, while in the rest of the species, the spur is robustly longer than the ovary. The column is well demarcated in all of the species with parallel anthers and stout caudicles; *H. josephi* and *H. glaucifolia* have parallel, closely lingulate stigmatic process; the stigma of *H. aitchisonii* are concave surrounding the opening of the spur.

**Figure 3.**
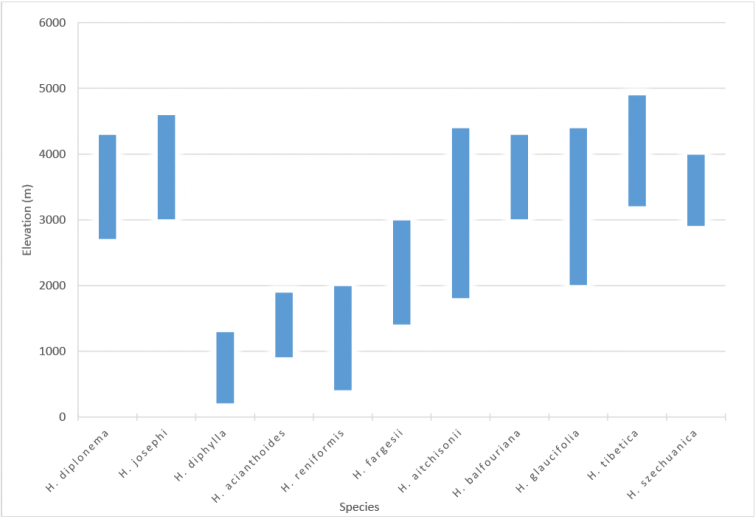
Altitudinal range of Habenaria sect. Diphyllae in the Pan-Himalaya.

**Ecology**: All species in the *Habenaria josephi* group are terrestrial herbs growing on moist grasslands, rocky surfaces and alpine gullies. Generally, they occur in the elevation range of 2000–5000 m (Fig. 3).

### Taxonomic synopsis

#### *Habenaria sect. Diphyllae* Kränzl., Bot. Jahrb. Syst. 16(2): 147. 1892.

##### Key to the species of *Habenaria*. sect. Diphyllae from the Pan-Himalaya

**Table:** 

1a	Plants 4–20 cm tall; petals entire	**2a**
2a	Rachis and ovary pubescent; sterile bracts absent.	**3a**
3a	Leaves glabrous; floral bracts ca. 10 mm long; ovary and pedicel 8–14 mm long; lateral lobes of lip linear with coiled tips, slightly longer than median lobe; spur 8–15 mm long	**1. *H. josephi***
3b	Leaves papillate; floral bracts ca. 3 mm long; ovary and pedicel ca. 7 mm long; lateral lobes of lip filiform with straight tips, much longer than median lobe; spur ca. 4 mm long	**2. *H. diplonema***
2b	Rachis and ovary glabrous; sterile bracts present.	**4a**
4a	Lip 3-lobed; spur distinct and always present.	**5a**
5a	Leaves usually 2; ovary ca. 10 mm long; petals falcate linear-lanceolate	**9. *H. diphylla***
5b	Leaf usually one; ovary 4–5 mm long; petals obliquely ovate	**10. *H. acianthoides***
4b	Lip simple, linear; spur indistinct or absent	**11. *H. reniformis***
1b	Plants 10–50 cm tall; petals distinctly 2-lobed.	**6a**
6a	Petals deeply 2-lobed, lower lobe linear to linear lanceolate, more than 4 mm long; lateral lobe of lip linear, with circinate tip.	**7a**
7a	Petal upper lobe falcate-oblong, ca. 4 mm long; lower lobe ca. 10 mm long; floral bracts ca. 7 mm long; ovary cylindric, 12–20 mm long	**3. *H. fargesii***
7b	Petal upper lobe spatulate-oblong, 12–15 mm long, lower lobe ca. 4 mm long; floral bracts ca. 15 mm long; ovary terete, 22–35 mm long	**4. *H. glaucifolia***
6b	Petals shallowly 2-lobed, lower lobe like a tooth, less than 2 mm long; lateral lobe of lip linear, retrorse, with bent, but not circinate tip.	**8a**
8a	Sepals 3–7 mm long, 2.5–4 mm broad; petals glabrous; spur reflexed, pendulous.	**9a**
9a	Inflorescence laxly to densely many flowered; ovary with pedicel 7–12 mm long; spur 7–8 mm long	**5. *H. aitchisonii***
9b	Inflorescence subdensely 3–12 flowered; ovary with pedicel 8–10 mm long; spur 12–20 mm long	**6. *H. balfouriana***
8b	Sepals 7–11 mm long, 3–5 mm broad; petals ciliate; spur spreading horizontally.	**10a**
10a	Leaves with green veins adaxially; lip base with a needle-like appendage near entrance of spur	**7. *H. szechuanica***
10b	Leaves with white veins adaxially; lip base lacking appendage near entrance of spur	**8. *H. tibetica***

**Note.** Of the 11 species of sect. Diphyllae from the Pan-Himalaya, only the eight species that belong to the high mountain clade (Jin et al. 2017, Fig. 3) are presented here.

##### 1. Habenaria josephi

Taxon classificationPlantaeAsparagalesOrchidaceae

Rchb. f., Trans. Linn. Soc. London, Bot., ser. 2, 3: 114 (1888).

6F925DE4-223E-5E35-8512-D3E0B97EC0DC

≡ Habenaria aitchisonii var. josephi (Rchb.f.) Hook.f., Fl. Brit. India 6: 152 (1890).
≡ Habenaria diphylla var. josephi (Rchb.f.) N. Pearce & P.J. Cribb, Edinburgh J. Bot. 58: 114. 2001. Type. INDIA, Sikkim, 1849, *J.D. Hooker 42* [holotype: K (K000247480 image!); isotypes: K, AMES (00256484 image!), P (P00370608 image!), LE n.v.].
*Habenaria clarkei* Kränzl., Bot. Jahrb. Syst. 16: 148 (1892). Type. INDIA, Sikkim, *J. D. Hooker 42* [lectotype designated here: K (K000247480 image!); isolectotypes: K, AMES (00256484 image!), P, LE n.v.].

###### Description.

**Figure 4.**
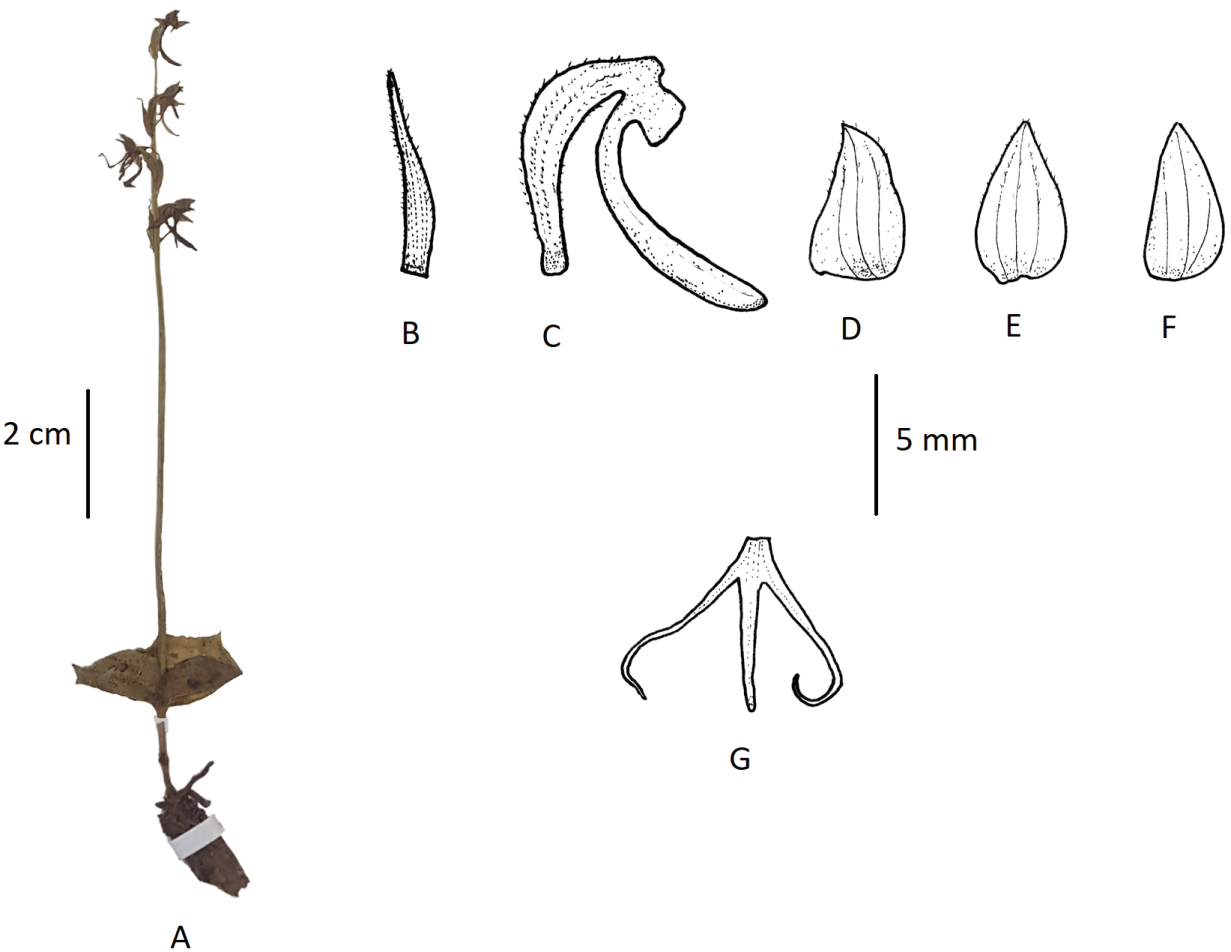
*Habenaria josephi***A** habit **B** floral bract **C** pedicellate ovary with spur **D** petal **E** dorsal sepal **F** lateral sepal **G** lip (**A** photographed from *FLPH Expedition 13-0845*, PE **B–G** drawn from the same specimen by T.R. Pandey).

Terrestrial herbs, 5–20 cm tall. Tubers ovoid-fusiform. Stems pubescent. Leaves 2, opposite, basal; sheathing at base; leaf blade broadly ovate-orbicular to weakly cordate, 1.5–3.1 cm long, 1–2.5 cm broad, apex apiculate. Inflorescences 4–15 cm long, laxly to subdensely 2- to 6-flowered; rachis minutely glandular, pubescent, 1.5–3.8 cm long; floral bracts narrowly lanceolate, ca. 1 cm long, pubescent, apex acuminate. Flowers green, fragrant; ovary and pedicel curved, 7–13 mm long, pubescent. Dorsal sepal ovate, 5.5–6 mm long, 2.5–3 mm broad, apex acute, forming hood with petals; lateral sepals ovate, reflexed to spreading, 5–7 mm long, ca. 2.6 mm broad, apex acute. Petals obliquely ovate-triangular, base broad, 5–6 mm long, 2–2.5 mm wide, apex acute; lip 3-lobed, clawed, spurred; lateral lobes linear, apex recurved-coiled, 6–9 mm long, ca. 0.6 mm wide; mid-lobe linear, ca. 5 mm long, ca. 0.5 mm broad; spur curved, clavate, 8–15 mm long. Column stout; anther locules parallel; pollinia globose-ovoid; caudicle stout; stigma processes closely parallel, united above mouth of spur, lingulate. (Fig. 4).

###### Phenology.

Flowering from July to September.

###### Habitat.

Moist grassy hillsides, stream banks, in *Betula* forest; 3000–4600 m elev.

###### Distribution.

**Figure 5.**
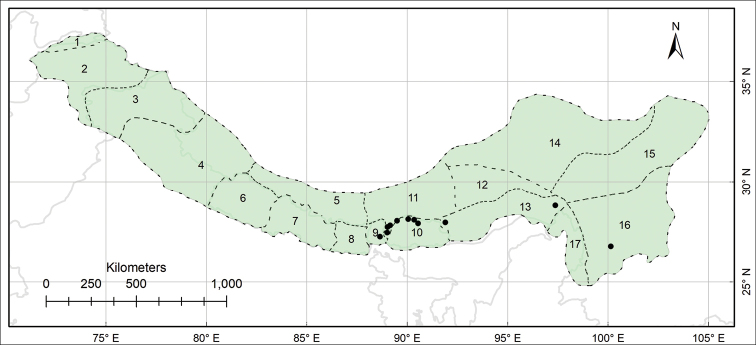
Distribution of *Habenaria josephi* (black circles) in the Pan-Himalaya.

Endemic to the Pan-Himalaya; Sikkim and Darjeeling, Bhutan, M Yarlung Zangbo, Yarlung Zangbo-Brahmaputra and S Hengduan. (Fig. 5).

###### More illustrations.

Pearce and Cribb (2002, fig. 41, a–m; as H. diphylla var. josephi).

###### Additional specimens examined.

**SIKKIM and DARJEELING: Sikkim**, Tungu, 3648–3952 m elev., 1849, *J.D. Hooker 42* (K). **BHUTAN: Bumthang**, Marlungm Tsampa, 4400 m elev., 1949, *F. Ludlow*, *G. Sherriff & J.H. Hicks 19397* (AMES 01946674); **Gasa**, Upper Mo Chu, 4120 m elev., 1984, *I.W.J. Sinclair & D.G. Long 5289* (RENZ); **Gasa**, Gafoo-la, Upper Pho Chu, 4000 m elev., 1949, *F. Ludlow*, *G. Sherriff & J.H. Hicks 16725* [CAL (CAL0000056823), AMES (01946675)]; **Jomolhari** (GLORIA site), 4514 m elev., 2010, *S. Den et al.* 193 (National Biodiversity Centre, Bhutan). **M YARLUNG ZANGBO: Yadong**, Phari, 1879, *Dungboo s. n.* [K (K000247481), CAL (CAL0000092705)]; **Yadong**, North of Phari, 1882, *Dr. King’s collectors s. n.* (CAL0000092702); **Yadong**, Tem-la, North of Phari, 1882, *G. King’s collectors s. n.* (CAL0000092703). **YARLUNG ZANGBO-BRAHMAPUTRA: Cona**, 3641 m elev., 2013, *FLPH Tibet Expedition 13-0957* (PE); **Zayü**, 4100 m elev., 2013, *FLPH Tibet Expedition 13-0845* (PE). **S HENGDUAN: Yulong**, eastern flank of Lichiang range, 3500 m elev., 1906, *G. Forrest 2739* (CAL0000055843).

###### Note.

Hooker (1890) tentatively placed some of the *Habenaria* species, such as *H. reniformis* (D. Don) Hook.f., *H. diphylla* Dalz. and *H. aitchisonii* Rchb.f., into the section Trimeroglossa. However, *H. josephi* was assigned a varietal rank under *H. aitchisonii*. Later, Kränzlin (1892, 1901) proposed the section Diphyllae to accommodate the species with two basal leaves and included *H. clarkei*, *H. glaucifolia*, *H. diphylla*, *H. reniformis* and *H. aitchisonii* in the section along with a few other *Habenaria* species. He followed Hooker’s view regarding the position of *H. josephi* as a variety of *H. aitchisonii*, albeit with a note “*Die var. Josephi Hook. f. ist nur eine Form*, *aus den höchsten der oben angegebenen Standorte stammend* (the variety *josephi* is only a form occurring in the highest of the above-mentioned locations)” suggesting that the plant is merely a higher elevation variety of *H. aitchisonii*. Paradoxically, Kränzlin described *H. clarkei* in the same publication, based on a duplicate of *Hooker 42* (the type specimen of *H. josephi*) from Sikkim, which was kept at B (distributed from Hooker’s Herbarium at K). After an extensive search, it now appears that *Hooker 42* had at least six duplicates: two at K and one each at P, AMES, B and LE, of which the specimen at B was lost during the Second World War, while at LE, the specimen could not be found during a recent search (fide Petr Efimov). The remaining four duplicates are still extant.

In the past, the taxonomic identity of *Habenaria josephi* became doubtful, often shifting from one name to another, sometimes as Habenaria aitchisoni var. josephi (Rchb.f.) Hook.f. or as H. diphylla var. josephi (Rchb.f.) Pearce & Cribb. Even in recent literature on the orchid species of Sikkim, its type locality is not uniform in this regard. Some botanists treat it as a variety of *H. diphylla* (Pearce et al. 2001; Pearce and Cribb 2002; Lucksom 2007; Choudhury et al. 2011), while Maity et al. (2019) regard it merely as another synonym of *H. diphylla*. A closer look at this species reveals it to be not only distinct morphologically, but also well characterised in terms of habitat and distribution. Whereas *H. diphylla* is predominantly a tropical species of moderate size (10–40 cm tall), broadly distributed from peninsular India to the Philippines, *H. josephi* is a small-sized (5–20 cm tall) high-elevation temperate to alpine species occurring from Sikkim eastwards to the Hengduan Mountains, i.e. it is endemic to the Pan-Himalaya. Pearce et al. (2001) provided an elaborate discussion on the phenetic variations that delineate this taxon from the other species, yet they assigned it to a varietal rank under *H. diphylla*. Examination of the type and other dried specimens, as well as living individuals, clearly shows that it is distinct. Short stature, scape without sterile bracts, 2–6-flowered rachis, curved, pubescent ovary, deflexed lateral sepals and lip with stooping (reflexed) lateral lobes that ultimately coil around terminally are amongst the unique diagnostic morphological features. Furthermore, recent molecular studies (Jin et al. 2017; Raskoti and Ale 2019) have consolidated its distinction from similar-looking species. Therefore, a specific rank seems fully justified here.

##### 2. Habenaria diplonema

Taxon classificationPlantaeAsparagalesOrchidaceae

Schltr., Notes Roy. Bot. Gard. Edinburgh 5: 100. 1912.

878DEE78-6B8C-535C-A020-607915744823

###### Type.

China, Yunnan, 3300–3600 m elev., 1906. *G. Forrest 2812* [holotype: E (E00381985 image!); isotypes: IBSC (0635875!), CAL (CAL0000000748!), P (P00426408 image!)].

###### Description.

**Figure 6.**
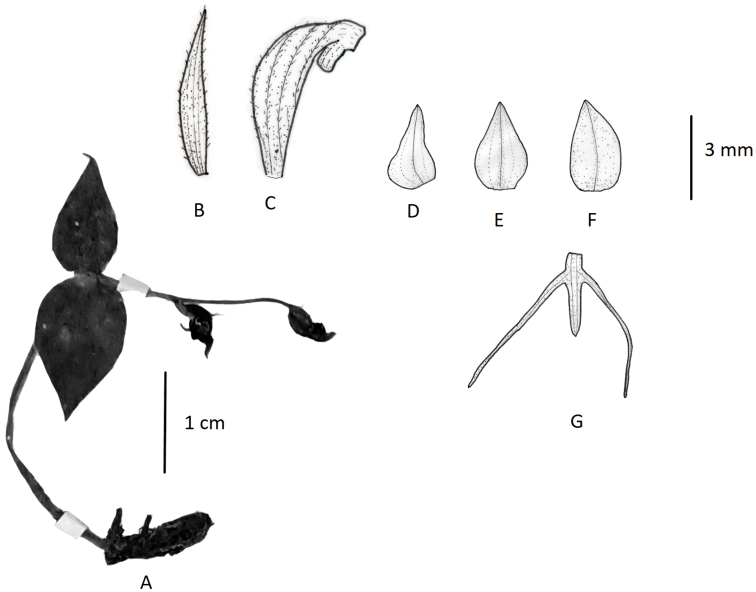
*Habenaria diplonema***A** habit **B** floral bract **C** pedicellate ovary with spur **D** petal **E** dorsal sepal **F** lateral sepal **G** lip (**A** photographed from *T.T. Yu 14014*, PE **B–G** drawn from the same specimen by T.R. Pandey).

Terrestrial herbs, 4–15 cm tall. Tubers globose-oblong. Stems densely papillate-pubescent. Leaves 2, opposite, basal; sheathing at base; leaf blade ovate to orbicular, 1–2.4 cm long, 1–2.2 cm broad, adaxially with yellowish-white venation, densely papillate, apex acute to acuminate. Inflorescence 3–12 cm long, sparsely 2–14-flowered; rachis 2–5.5 cm long, pubescent; floral bracts lanceolate, 3–6 mm long, apex acuminate. Flowers green, faintly fragrant; ovary and pedicel curved, 6–8 mm long, pubescent. Dorsal sepal broadly ovate, ca. 3.5 mm long, ca. 3 mm broad, glabrous, apex obtuse; lateral sepals oblique, ovate-elliptic, deflexed, ca. 4 mm long, ca. 2.5 mm broad, glabrous, apex obtuse. Petals obliquely falcate-ovate, ca. 3.5 mm long, 2–2.5 mm broad, glabrous, entire; lip 3-lobed, spurred; lateral lobes filiform, ca. 10 mm long; mid-lobe linear-lingulate, ca. 3 mm long; spur pendulous, clavate, 1–4 mm long. Column short; anther apex retuse; caudicles short; stigma processes clavate. (Fig. 6).

###### Phenology.

Flowering from July to September.

###### Habitat.

Shady cliffs and rocks; 2700–4300 m elev.

###### Distribution.

**Figure 7.**
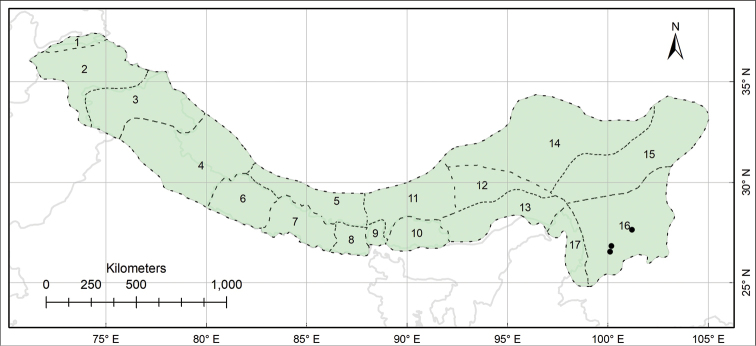
Distribution of *Habenaria diplonema* (black circles) in the Pan-Himalaya.

S Hengduan; also in N Fujian of China. (Fig. 7).

###### More illustrations.

Wu et al. (2010, fig. 192, 7–9).

###### Additional specimens examined.

**S. HENGDUAN: Muli**, Rangetzantze, 3500 m elev., 1937, *T.T. Yü 14014* (KUN, PE); **Yulong (Lijiang)**, 2800 m elev., 1935, *C.W. Wang 70748* (PE, KUN); **Yulong (Lijiang)**, 4200 m elev., 1914, *C. Schneider 2459* (K).

###### Note.

The photograph of the type specimen housed at the herbarium of Royal Botanic Garden Edinburgh (*G. Forrest 2812*, E00381985) was published along with the protologue, therefore this specimen is the holotype (Art. 9.1, Note 1 (b), Turland et al. 2018).

##### 3. Habenaria fargesii

Taxon classificationPlantaeAsparagalesOrchidaceae

Finet, Rev. Gen. Bot. 13: 528, t. 18A. 1–8. 1901.

9D33FDB1-B138-5CE7-8805-7193BAC074BA

###### Type.

China, Sichuan, 1400 m elev., 1893, *Farges 1279* [lectotype designated here: P (P00426411 image!)].

###### Description.

**Figure 8.**
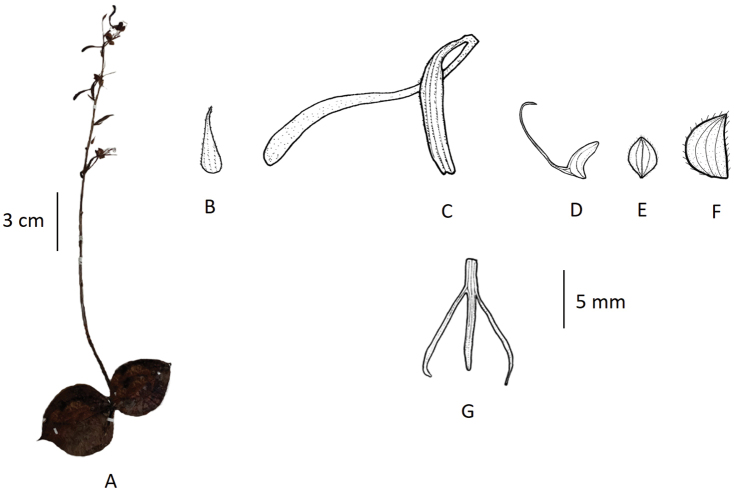
*Habenaria fargesii***A** habit **B** floral bract **C** pedicellate ovary with spur **D** petal **E** dorsal sepal **F** lateral sepal **G** lip (**A** photographed from *FLPH Sichuan Expedition 152179*, PE **B–G** drawn from the same specimen by T.R. Pandey).

Terrestrial herbs, 13–37 cm tall. Tubers ovoid or oblong. Stems erect or ascending, finely papillate-pubescent. Leaves 2, opposite, basal; base narrowed and amplexicaul; leaf blade spreading horizontally, adaxially yellowish-white marked, ovate or orbicular, 4–6 cm long, 3.5–6 cm broad, apex acute. Inflorescence 10–30 cm long, 4–9-flowered; rachis 5–11 cm long, finely papillate-pubescent; floral bracts lanceolate, ca. 7 mm long, apex acuminate. Flowers yellowish-green; ovary and pedicel twisted, 1.2–2 cm long, pubescent. Dorsal sepal ovate, 3–3.5 mm long, ca. 2.5 mm broad, margins ciliate, apex acute; lateral sepals strongly reflexed, obliquely ovate, 5–7 mm long, ca. 4 mm broad, margins ciliate, apex acute. Petals connivent with dorsal sepal, deeply 2-lobed; upper lobe falcate-oblong, ca. 4 mm long; lower lobe linear, ca. 10 mm long; lip deeply 3-lobed above the base, spurred; lateral lobes divaricate, filiform, ca. 1.5 cm long, apex curled; mid-lobe linear, ca. 1 cm long; spur pendulous, 2–2.5 cm long. Column short and broad, anther connective wide; pollinia granular; caudicles linear; stigmatic processes elongated. (Fig. 8).

###### Phenology.

Flowering in July to September.

###### Habitat.

Montane forests, grassy valleys; 1400–3000 m elev.

###### Distribution.

**Figure 9.**
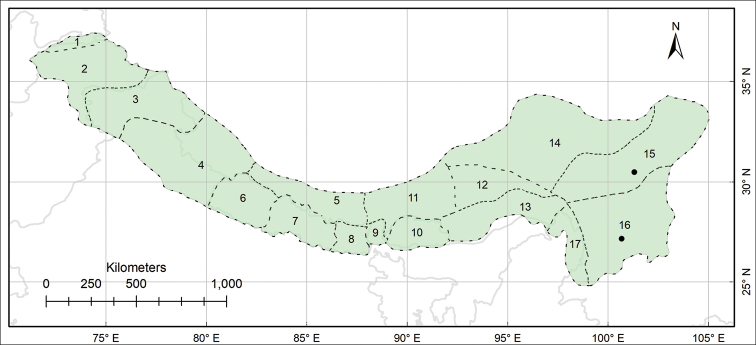
Distribution of *Habenaria fargesii* (black circles) in the Pan-Himalaya.

N and S Hengduan; also in Chongqing and Gansu of China (Fig. 9).

###### More illustrations.

Finet (1901, fig. 18A, 1–8).

###### Additional specimens examined.

**N HENGDUAN**: Sichuan, *T.T. Li 644* (PE). **S HENGDUAN: Yanyuan**, 3000 m elev., 2015, *FLPH Sichuan Expedition 152179* (PE).

##### 4. Habenaria glaucifolia

Taxon classificationPlantaeAsparagalesOrchidaceae

Bureau & Franch., J. Bot. (Morot) 5: 152. 1891.

7AE48869-4233-5F29-81D6-80E079DF5A8E

≡ *Senghasiella glaucifolia* (Bureau & Franch.) Szlach. J. Orchideenfreund 8: 365. 2001.

###### Type.

China, Sichuan, *Prince Henry D’Orleans s. n.* [lectotype designated here: P (P00426784 image!)].

###### Description.

**Figure 10.**
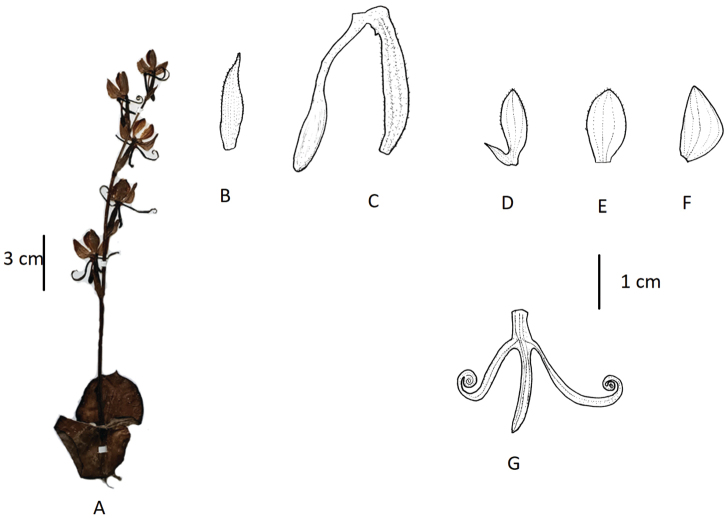
*Habenaria glaucifolia***A** habit **B** floral bract **C** pedicellate ovary with spur **D** petal **E** dorsal sepal **F** lateral sepal **G** lip (**A** photographed from *K.Y. Lang et al. 945*, PE **B–G** drawn from the same by T.R. Pandey).

Terrestrial herbs, 12–50 cm tall. Tubers oblong or ovoid. Stems erect, pubescent. Leaves 2, opposite, basal; base obtuse-rounded and amplexicaul; leaf blade spreading horizontally, abaxially tinged with greyish-white, adaxially purplish-green, ovate-orbicular, 2–5 cm long, 1–4.7 cm broad, apex acute-acuminate. Inflorescence 8–45 cm long, 2–8-flowered; rachis 4–18 cm long, pubescent; floral bracts lanceolate or ovate, apex acuminate. Flowers yellowish to yellowish-white; ovary and pedicel twisted, 2.2–3.5 cm long, pubescent. Dorsal sepal forming a hood with petals, erect, ovate or oblong, concave, 10–13 mm long, 6–7 mm broad, apex obtuse; lateral sepals reflexed, obliquely ovate or oblong, 11–14 mm long, 7–7.5 mm broad, apex acute. Petals deeply 2-lobed; upper lobe spatulate-oblong, 12–15 mm long, ca. 6 mm broad, margin ciliate, apex obtuse; lower lobe linear-lanceolate, ca. 4 mm long, ca. 1 mm broad, apex acute; lip reflexed, base with a short claw, deeply 3-lobed, spurred; lateral lobes divaricate, linear, 2.5–4 cm long, circinate toward apex; mid-lobe straight, linear, 1.1–1.4 cm long; spur pendulous, cylindrical-subclavate, 2–3 cm long. Column short; anther parallel, connective wide; pollinia granular; caudicles slender, elongated; stigmatic processes closely parallel, lingulate. (Fig. 10).

###### Phenology.

Flowering from July to September.

###### Habitat.

Montane forests, grasslands; 2000–4400 m elev.

###### Distribution.

**Figure 11.**
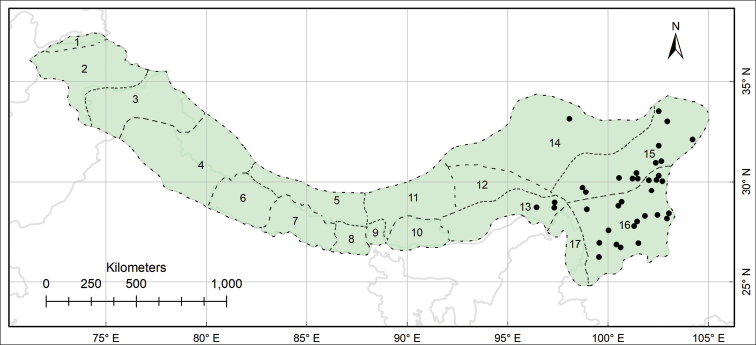
Distribution of *Habenaria glaucifolia* (black circles) in the Pan-Himalaya.

Yarlung Zangbo-Brahmaputra, Tangut, N and S Hengduan; widely spread in eastern part of Pan-Himalayan Region. Also found in Gansu, Guizhou and Shaanxi of China. (Fig. 11).

###### More illustrations.

Wu et al. (2010, fig. 194, 1–2).

###### Additional specimens examined.

**YARLUNG ZANGBO-BRAHMAPUTRA: Mishmi Hills**, 1911, *Bailey s. n.* (E); **Zayü**, Guyu, Luoma, 2996 m elev., 2009, *X.H. Jin et al. SET-ET344* (PE); **Zayü**, Shang, 3400 m elev., 2013, *Jin et al. ST-2554* (PE). **TANGUT: Sêrxü**, 3900 m elev., 1974, *Vegetation Team of Sichuan 5771* (PE). **N HENGDUAN: Danba**, 3000 m elev., 1940, *Qu 7523* (PE); **Heishui**, 3200 m elev., 1957, *Li 73181* (PE); **Heishui**, 1959, *Chuan 1432* (PE); **Hongyuan**, 2900 m elev., 1957, *Zhang & Zhou 22665* (PE); **Jinchuan**, 2450 m elev., 1957, *Li 75398* (PE); **Litang-Yalong**, 1921, *F. Kingdon-Ward 4466* (E); **Maoxian**, 1952, *He & Zhou 13230* (PE); **Markam**, 2700 m elev., 1957, *s. coll. 22969* (PE); **Markam**, 2900 m elev., *W.L. Chen 766* (PE); **Xiaojin**, Gasiling, 3100 m elev., 1957, *J. Zhou 204* (IBSC); **Xiaojin**, 2450 m elev., 1958, *Zhang & Wang 5907* (PE); **Yajiang**, 3700 m, elev.1961, *Jiang 9863* (PE); **Yajiang**, 2875 m elev., 2006, *Boufford et al.* 35947 (PE, KUN). **S HENGDUAN: Daocheng**, 3100 m elev., 1973, *Sichuan Vegetation Survey Team 2391* (PE): **Daocheng**, 4300 m elev., 1973, *Sichuan Vegetation Survey Team 3645* (PE): **Dêqên**, 3100 m elev., 1937, *T.T. Yü 9867* (PE); **Eryuan**, 2900 m elev., 1963, *NW Yunnan Team 6339* (PE); **Ganluo**, 2000 m elev., 1959, *Chuan 4191* (PE); **Kangding**, 1951, *Hu & He 10401* (PE); **Kangding**, 3500 m elev., 1963, *Guan & Wang 808* (PE); **Kangding**, 3650 m elev., 1982, *K.Y. Lang et al. 945* (PE); **Luding**, 2420 m elev., 1959, *Jiang & Jin 1949* (PE); **Meigu**, 2100 m elev., 1959, *Chuan 1113* (PE); **Meigu**, 2300 m elev., 1976, *s. coll. 13083* (PE); **Mianning**, 3300 m elev., 1940, *Qu 7353* (PE); **Muli**, 1921, *F. Kingdon-Ward 4571* (E); **Muli**, Ye-tze, 3100 m elev., 1937, *T.T. Yü 7022* (PE); **Shangri-la**, 3200 m elev., 2010, *Kham Expedition 10-3079* (PE); **Tianquan**, 1959, *Chuan 853* (PE); **Weixi**, 3200 m elev., 1935, *C.W. Wang 68011* (PE); **Yanyuan**, 2500–2600 m elev., 1960, *Jiang 5991* (PE); **Yulong (Lijiang)**, 2900 m elev., 1981, *Hengduanshan Team of Beijing Institute 02666* (PE); **Yulong (Lijiang)**, 1910, *G. Forrest 6050* (PE).

##### 5. Habenaria aitchisonii

Taxon classificationPlantaeAsparagalesOrchidaceae

Rchb.f., Trans. Linn. Soc. London, Bot., ser. 2, 3: 113. 1888.

88FAC9E9-E7BE-57F2-BE5A-37875F3CE058

*Habenaria wolongensis* K.Y. Lang, Acta Phytotax. Sin. 22(4): 314, 1984, syn. nov. Type. CHINA, Sichuan, Wolong, 2200 m elev., 1982, *K. Y. Lang*, *L. Q. Li & Y. Fei 1528* [lectotype designated by Pandey and Jin (in press): PE (01147127!), isolectotype: PE (01147128!)].
*Habenaria diceras* Schltr., Notes Roy. Bot. Gard. Edinburgh 5: 101, t. 78. 1912. Type. CHINA, Yunnan, Lijiang Range eastern flank, 2700–3000 m elev., 1906, *G. Forrest 3074* [holotype: E (E00381986 image!), isotype: P (P00426380 image!].
*Habenaria bihamata* Kränzl., Repert. Spec. Nov. Regni Veg. 17: 106. 1921. Type. CHINA, Yunnan, Pe yen tsin, *S. Ten s. n.* (type: B n.v., probably lost).
*Habenaria pubicaulis* Schltr., Acta Horti Gothob. 1: 139. 1924. Type. CHINA, Sichuan, ca. 3900 m elev., 1922, *Harry Smith 3858* [holotype: UPS (V-091292 image!); isotypes: PE (01516965!), E (E00381983 image!), LD (1073030 image!), S (S07-285 image!)].

###### Type.

Afghanistan (now Pakistan), Darban Valley, Kuram District, 2280 m elev., 1880, *Aitchison 413* [holotype: K (K000247484 image!), isotype: AMES (00256482 image!)].

###### Description.

**Figure 12.**
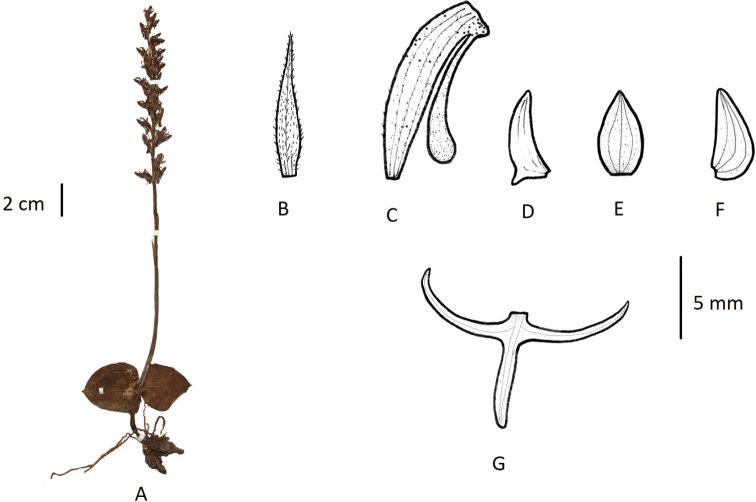
*Habenaria aitchisonii***A** habit **B** floral bract **C** pedicellate ovary with spur **D** petal **E** dorsal sepal **F** lateral sepal **G** lip (**A** photographed from *K.Y. Lang et al. 944*, PE **B–G** drawn from the same specimen by T.R. Pandey).

Terrestrial herbs, 10–50 cm tall. Tubers oblong or ellipsoid. Stems erect, papillate-pubescent. Leaves 2, opposite, basal; base narrowed and amplexicaul; blade ovate or ovate-orbicular, 2–7 cm long, 1.5–6 cm broad, apex acute. Inflorescence 8–45 cm long, laxly to densely many (up to 40) flowered; rachis 1.5–8 cm long, papillate-pubescent; sterile bracts ovate to lanceolate, 0.7–1.5 cm long, acuminate; floral bracts ovate-lanceolate, margins ciliate, ca. 0.7 cm long, apex acuminate. Flowers bright green, with often faintly yellowish lip; ovary and pedicel arcuate, 0.7–1.2 cm long, papillate-hairy. Dorsal sepal forming a hood with petals, erect, ovate, concave, 3–5 mm long, 2.5–3.5 mm broad, apex obtuse or acute; lateral sepals reflexed, obliquely ovate-oblong, 3.5–5.5 mm long, 2.5–3 mm broad, apex obtuse or acute. Petals 2-lobed, glabrous; upper lobe obliquely falcate-lanceolate, 3–5 mm long, 1.5–2 mm broad; lower lobe a tooth at the base of upper lobe, ca. 0.5 mm long; lip deeply 3-lobed near the base, spurred; lateral lobes linear, retrorse, embracing erect sepals, 6–12 mm long, apex slightly bent; mid-lobe reflexed, linear, 5–9 mm long, ca. 1 mm broad; spur pendulous, cylindrical-clavate, 7–8(-10) mm long. Column stout, anthers diverging, connective wide; pollinium granular; caudicles short, stout; stigmatic processes converging, subclavate. (Fig. 12).

###### Phenology.

Flowering from July to September.

###### Habitat.

Open *Juniperus*/*Larix* montane forests, thickets, grasslands; 1800–4400 m elev.

###### Distribution.

**Figure 13.**
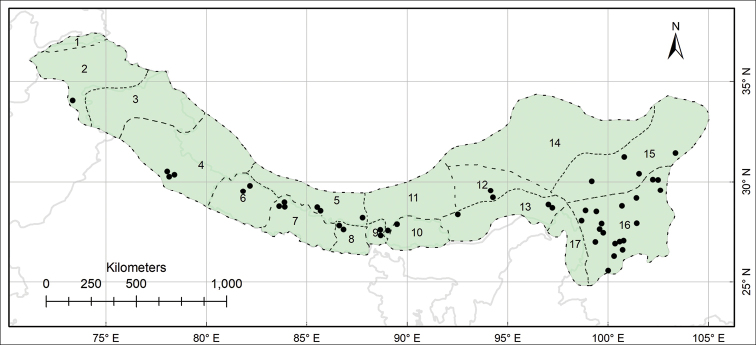
Distribution of *Habenaria aitchisonii* (black circles) in the Pan-Himalaya.

N Pakistan, U Ganga and Indus, U Yarlung Zangbo, W, C and E Nepal, Sikkim and Darjeeling, Bhutan, M Yarlung Zangbo, L Yarlung Zangbo, Yarlung Zangbo-Brahmaputra, N and S Hengduan. (Fig. 13).

###### More illustrations.

Wu et al. (2010, fig. 192, 4–6).

###### Additional specimens examined.

**N PAKISTAN: Hazara**, Rawalpindi district, 2500 m elev., 1975, *J. Renz 10800* (RENZ). **U GANGA & INDUS**: **Mussoorie**, 2128 m elev., 1898, *P.W. Mackinnon s. n.* (CAL0000092710); **Mussoorie**, 1824 m elev., 1899, *P.W. Mackinnon 22991* (CAL0000092691); **Mussoorie**, 2300 m elev., 1983, *J. Renz & Y.P.S. Pangtey 13641* (RENZ). **U YARLUNG ZANGBO**: **Dinggyê**, 4151 m elev., 2013, *PE-Tibet team 3421* (PE); **Gyirong (Jilong)**, 3700 m elev., 2013, *PE-Tibet team 3825* (PE); **Gyirong (Jilong)**, 2950 m elev., 1975, *Qinghai-Tibet Team 7034* (PE). **W NEPAL**: **Karnali**, Mugu, Pina-Ghurchi, 2800 m elev., 1985, *P.R. Shakya*, *M.N. Subedi*, *R.K. Uprety 8783* (KATH); Jumla to Dori Lekh, 3200 m elev., 1979, *K.R. Rajbhandari & B.Roy 3378* (KATH). **C NEPAL**: **Dhawalagiri**, Mustang, Muktinath-Thorungse, 3900 m elev., 1983, *K.R. Rajbhandari 8173* (KATH); Mustang, Dhampus, 2450 m elev., 1988, *M. Suzuki et al. 8881592* (KATH); Mustang, Larjung, 2550 m elev., 1996, *T. Hoshino et al. 9668062* (KATH); Mustang, 1999, *S. Ishizawa et al. 990912020* (TI). **E NEPAL**: **Sagarmatha**, Solukhumbu, 3570 m elev., 2005, *Watson et al. DNEP3 BX107* (KATH); Solukhumbu, Thame, 2800 m elev., 2013, *B.B. Raskoti 00987* (KATH). **SIKKIM and DARJEELING**: **Sikkim**, Lachen Valley, 3648 m elev., 1895, *R. Pantling 398* (CAL); **Lachen**, 2584 m elev., 1909, *Smith & Cave 2669* (CAL0000092699). **BHUTAN**: **Gasa**, Upper Mo Chu, Laya, 3950 m elev., 1983, *C. Sargent 114* (RENZ 16003). **M YARLUNG ZANGBO**: **Yadong**, Phari, Kang me, 1882, *Dr. King’s Collector s. n.* (CAL0000092702). **L YARLUNG ZANGBO**: **Mainling**, 3100 m elev., 1972, *Tibet Chinese Medicinal Plants Survey team 3858* (PE); **Nyingchi**, near township, 3200 m elev., 1964, *Anonymous 793* (PE). **YARLUNG ZANGBO-BRAHMAPUTRA**: **Lhünzê**, 3889 m elev., 2013, *FLPH Tibet Expedition 13-0890* (PE); **Zayü**, 4100 m elev., 1973, *Qinghai-Tibet Team 1218* (PE); **Zayü**, 3200 m elev., 1935, *C.W. Wang 65772* (PE). **N HENGDUAN**: **Batang**, 3520 m elev., 1983, *K.Y. Lang et al. 2410* (PE); **Danba**, 3000 m elev., *G.L. Qu 7522* (PE); **Luhuo Xian**, NW of Luhuo along highway 317, 3385 m elev., *D.E. Bufford et al. 33485* (K); **Wenchuan**, Wolong Nature Reserve, 2220 m elev., 1982, *Lang et al. 1528* (type of *H. wolongensis*, PE). **S HENGDUAN**: **Cawarong**, 3450 m elev., 1982, *Qinghai-Tibet Team 10547* (PE); **Dali**, 3000 m elev., 2001, *H.K. Kadoorie Team s. n.* (PE); **Daocheng**, 3100–3200 m elev., 1981, *Qinghai-Tibetan Team 4235* (PE); **Dêqên**, 2200 m elev., 1935, *C.W. Wang 69920* (PE); **Heishui**, 3200 m elev., *Q. Li 73191* (PE); **Heqing**, 2730 m elev., 1996, *Y.B. Luo 40* (PE); **Jinyang**, 3500 m elev., *K.Y. Lang 14900* (PE); **Jiulong**, Tanggu Xiang, 3700 m elev., 2005, *D.E. Bufford 33420* (K); **Kangding**, 3470 m elev., 1982, *Lang et al. 1534* (PE); **Kangding**, 3650 m elev., 1982, *K.Y. Lang et al. 944* (PE); **Luding**, 2500 m elev., 1938, *Z.B. Wang 9772* (PE); **Muli**, 3200–3400 m elev., 1983, *Qinghai-Tibet Team 14063* (PE); **Shangri-la**, 4200–4300 m elev., 1981, *Hengduan Team 3322*, *2832* (PE); **Shangri-la**, Napa Hai, amongst shrubs, 3350 m elev., 2002, *H. Sun 08* (K); **Shangri-la** (Zhongdian), 3000 m elev., 1937, *T.T. Yu 12539* (PE); **Weixi**, 3600 m elev., 1935, *C.W. Wang 68356* (PE); **Xiangcheng**, 3500 m, elev. *Qinghai-Tibetan Team 004810* (PE); **Yulong (Lijiang)**, 3040 m elev., 1922, *G. Forrest 22208* (K); **Yulong (Lijiang)**, 2700–3000 m elev., 1913, *G. Forrest 10985* (PE); **Yulong (Lijiang)**, 2750 m elev., 1981, *Qinghai-Tibet Team 213* (PE).

###### Note.

This species is distributed along the whole range of the Himalaya up to the Hengduan Mountains at elevations between 2000 and 4500 m (temperate to alpine) and thus is the most widespread *Habenaria* species in the Pan-Himalaya. The type material was collected from the Darban Valley along the Pakistan-Afghanistan border in the western end of the distribution (Aitchison 1888). Similar plants from the Hengduan Region were described as new species by Schlechter (1912, 1924) and Kränzlin (1921), respectively, but were later reduced to synonyms (Chen and Cribb 2009). There is considerable variation in the size of the plant, in the colouring of the leaves, with yellowish-white markings that occasionally give a false impression of this being a new species; the peduncle is also quite variable in length as is the density of the inflorescence, from subdensely few-flowered to densely many-flowered. *Habenaria wolongensis* from Sichuan also falls within the range of variation of *H. aitchisonii* and is here reduced to synonymy.

##### 6. Habenaria balfouriana

Taxon classificationPlantaeAsparagalesOrchidaceae

Schltr., Repert. Spec. Nov. Regni Veg. 20: 381 (1924).

5E0C77F0-CF94-540C-8752-101BF24E4BD6

###### Type.

China, Yunnan, 1910, *G. Forrest 6149* [lectotype designated here: E (E00381989 image!); isolectotypes: PE (00340644!), IBSC (0636129!), K (K000796932 image!), P (P00370551 image!)].

###### Description.

**Figure 14.**
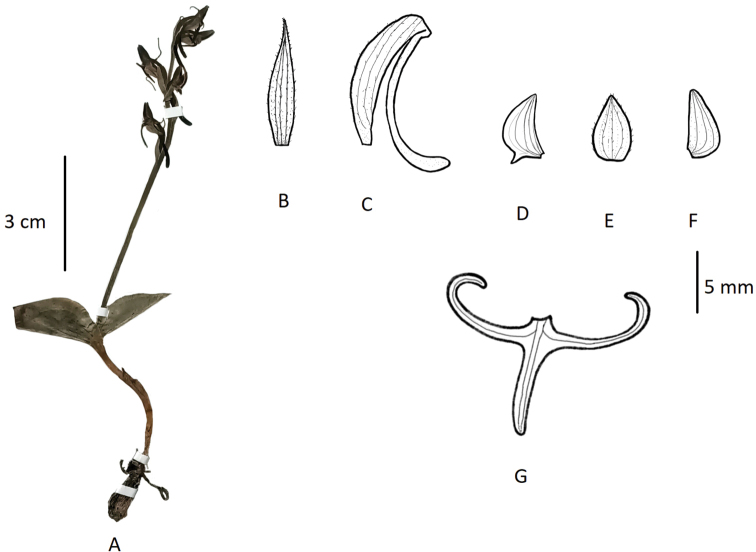
*Habenaria balfouriana***A** habit **B** floral bract **C** pedicellate ovary with spur **D** petal **E** dorsal sepal **F** lateral sepal **G** lip (**A** photographed from the isolectotype specimen *G. Forrest 6149*, PE **B–G** drawn from the same specimen by T.R. Pandey).

Terrestrial herbs, 10–24 cm tall. Tubers oblong. Stems densely pubescent. Leaves 2, opposite, basal; leaf blade ovate or ovate-orbicular, 2–4.5 cm long, 2–4 cm broad, fleshy, apex acuminate or acute. Inflorescence 8–20 cm long, subdensely 3–12-flowered; rachis 5–10 cm long; floral bracts lanceolate, apex acuminate. Flowers yellowish-green; ovary and pedicel arcuate, fusiform, 0.8–1 cm long, finely papillate-hairy. Dorsal sepal forming a hood with petals, erect, ovate, concave, 5–6 mm long, 3.5–4 mm broad, margin ciliate-denticulate, apex obtuse; lateral sepals oblique, ovate-oblong, reflexed, 6–7 mm long, 3.5–4 mm broad, apex subacute. Petals 2-lobed, glabrous; upper lobe obliquely ovate-lanceolate, 5–6 mm long, 2–2.2 mm broad; lower lobe a tooth at base of upper lobe, ca. 0.5 mm long; lip deeply 3-lobed above base, spurred; lateral lobes linear, retrorse, almost embracing ovary, linear, 1–1.2 cm long, apex bent; mid-lobe linear, reflexed, ca. 1 cm long; spur pendulous, slightly curved, clavate, 1.2–2 cm long. Column stout, anthers parallel, connective wide; stigmatic processes sub-oblong. (Fig. 14).

###### Phenology.

Flowering in July and August.

###### Habitat.

Montane forests, shrubby grasslands, alpine meadows; 3000–4300 m elev.

###### Distribution.

**Figure 15.**
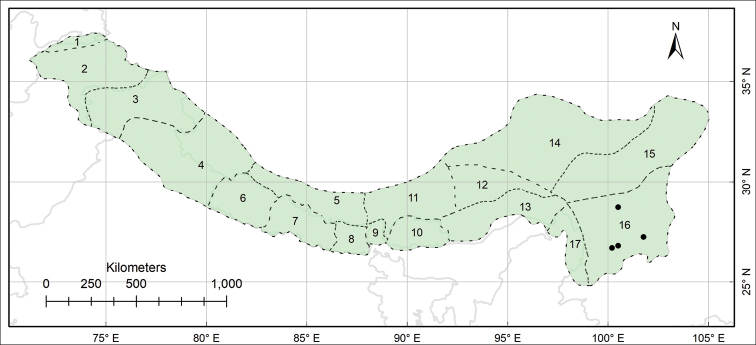
Distribution of *Habenaria balfouriana* (black circles) in the Pan-Himalaya.

Endemic to the Pan-Himalaya, found only in Hengduan Mountains; S Hengduan. (Fig. 15).

###### More illustrations.

Wu et al. (2010, fig. 194, 3–4).

###### Additional specimens examined.

**S HENGDUAN**: **Daocheng**, 4236 m elev., 2007, *X.H. Jin 9194* (PE); **Yanyuan**, 3600 m elev., 1983, *Qinghai-Tibet Team 12529* (PE); **Yulong (Lijiang)**, 3000 m elev., 1937, *T.T. Yu 15316* (PE); **Yulong (Lijiang)**, mountain meadows, 3344 m elev., 1906, *G. Forrest 2739* (K).

###### Note.

This species grows above 3000 to 4300 m in grassy alpine meadows. *Habenaria balfouriana* has a restricted distribution in the southern Hengduan Mountains, sharing the habitat with the similar-looking *H. aitchisonii*. Though *H. aitchisonii* and *H. balfouriana* were found closely allied in a recent molecular study (Jin et al. 2017), they are distinct morphologically; both have yellowish-green flowers with bilobed petals, but the former has an elongated spur that exceeds the length of the ovary and pedicel (versus spur shorter than ovary in *H. aitchisonii*).

##### 7. Habenaria szechuanica

Taxon classificationPlantaeAsparagalesOrchidaceae

Schltr., Acta Horti Gothob. 1: 140. 1924.

AE76594F-764B-571B-A30C-F6E03C872CC9

###### Type.

China, Sichuan, 3200 m elev., 1922, *Harry Smith 2916* [holotype: UPS (UPS-V-109140 image!); isotypes: E (E00381982 image!), LD (1072390 image!), PE (01516964!), S (S-G-7344 image!)].

###### Description.

**Figure 16.**
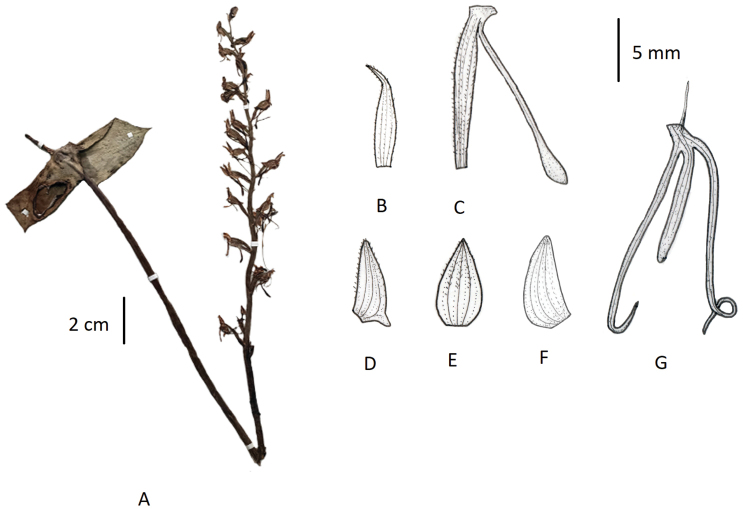
*Habenaria szechuanica***A** habit **B** floral bract **C** pedicellate ovary with spur **D** petal **E** dorsal sepal **F** lateral sepal **G** lip (**A** photographed from *Hengduan Mountain Team 02687*, PE **B–G** drawn from the same specimen by T.R. Pandey).

Terrestrial herbs, 18–30 cm tall. Tubers subglobose or ellipsoid. Stems erect, finely papillate-hairy. Leaves 2, opposite, basal; base obtuse-rounded, abruptly narrowed and amplexicaul; leaf blade broadly ovate or orbicular, 3–5 cm long, 3–5 cm broad, apex shortly acuminate or acute. Inflorescence 15–26 cm long, 3–8-flowered; rachis 5–12 cm long, papillate-hairy; floral bracts linear or lanceolate, apex acuminate. Flowers yellowish-green; ovary and pedicel twisted, fusiform, 1.2–1.5 cm long, papillate-hairy. Dorsal sepal forming a hood with petals, erect, ovate, concave, 7–8 mm long, 3–4 mm broad, apex obtuse; lateral sepals reflexed, obliquely ovate, 7–9 mm long, 3.5–4 mm broad, apex subobtuse. Petals shallowly 2-lobed; upper lobe obliquely oblong-lanceolate, 7–9 mm long, 2–2.5 mm broad, margin papillate-ciliate, apex obtuse; lower lobe a tooth at the base of upper lobe, ca. 1.5 mm long; lip reflexed, spurred, adaxially with a needle-like 5–7 mm long appendage near the entrance of spur, deeply 3-lobed; lateral lobes linear-filiform, 2.4–2.8(–4) cm long, apex often curled; mid-lobe linear, 1.2–2 cm long; spur cylindrical-clavate, 1.6–2.4 cm long, horizontally curved. Column stout, anther resupinate; stigma processes narrow, lingulate. (Fig. 16).

###### Phenology.

Flowering in July and August.

###### Habitat.

Montane forests with *Pinus yunnanensis* Franch. and *Picea* sp.; 2900–4000 m elev.

###### Distribution.

**Figure 17.**
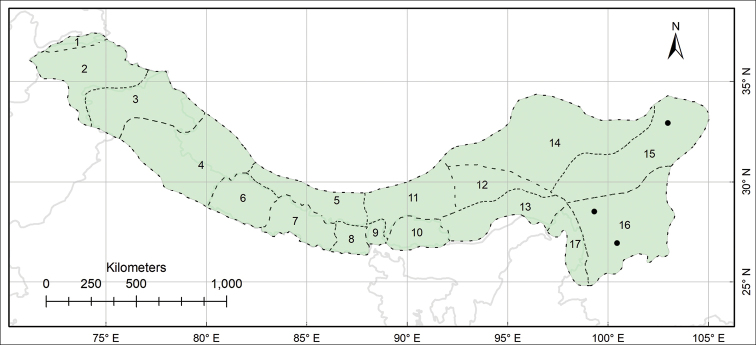
Distribution of *Habenaria szechuanica* (black circles) in the Pan-Himalaya.

N and S Hengduan; also in Shaanxi of China. (Fig. 17).

###### More illustrations.

Wu et al. (2010, fig. 195, 1–4).

###### Additional specimens examined.

**N HENGDUAN**: **Songpan**, 3450 m elev., 2002, *Y.B. Luo 850* (PE). **S HENGDUAN**: **Xiangcheng**, 3900 m elev., 1981, *Team of Qinghai-Tibetan Plateau 4782c* (PE). **Yulong (Lijiang)**, 2900 m elev., 1981, *Hengduan Mountain Team 02687* (PE, 4 duplicates).

##### 8. Habenaria tibetica

Taxon classificationPlantaeAsparagalesOrchidaceae

Schltr. ex Limpricht, Repert. Spec. Nov. Regni Veg. Beih. 12: 338. 1922.

AA44C41C-CE53-53F2-84FA-9C353CD90601

###### Type.

**Figure 18.**
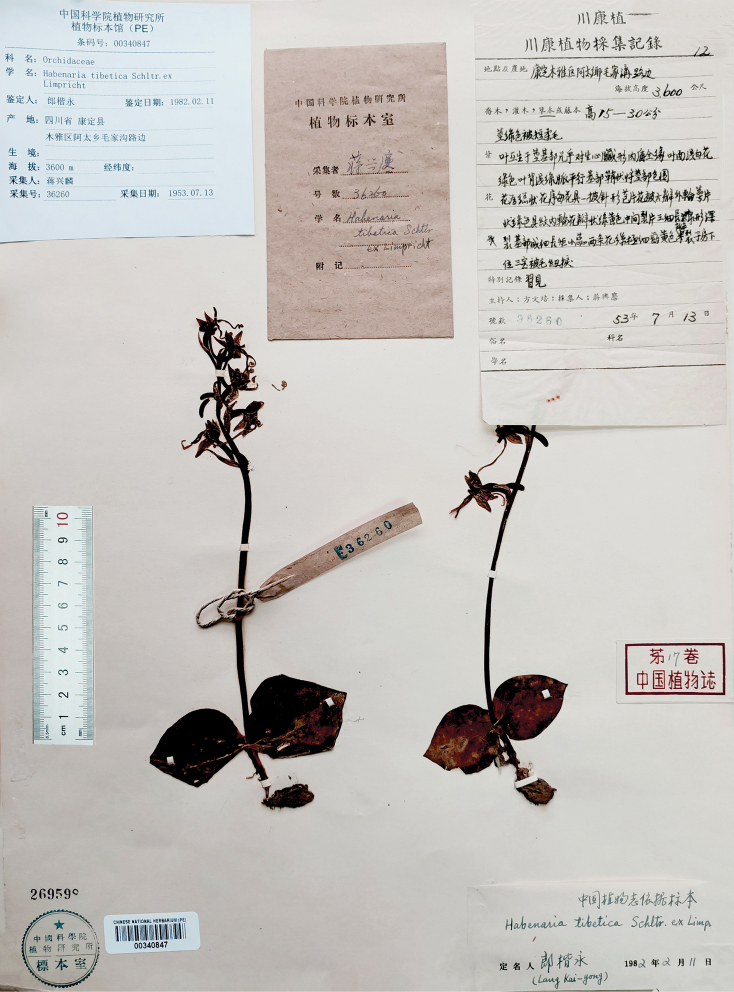
Neotype of *Habenaria tibetica* Schltr. ex Limpricht *X.L. Jiang 36260* (PE 00340847) (Photographed by T.R. Pandey).

China, Kangding, 3600 m elev., 13 July 1953, *X.L. Jiang 36260* (neotype designated here: PE 00340847!) (Fig. 18).

###### Description.

**Figure 19.**
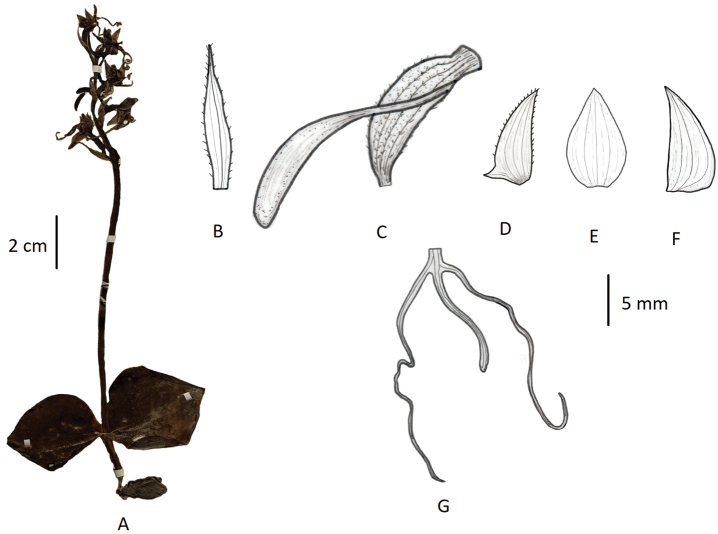
*Habenaria tibetica***A** habit **B** floral bract **C** pedicellate ovary with spur **D** petal **E** dorsal sepal **F** lateral sepal **G** lip (**A** photographed from the neotype *X.L. Jiang 36260*, PE **B–G** drawn from the same specimen by T.R. Pandey).

Terrestrial herbs, 12–40 cm tall. Tubers narrowly oblong to ellipsoid. Stems mostly underground, papillate-hairy. Leaves 2, nearly opposite, basal; prominent white veins on adaxial surface, base abruptly narrowed and amplexicaul; leaf blade wide ovate or orbicular, 2.5–6.5 cm long, 2.5–7 cm wide, apex obtuse or acute. Inflorescence 10–35 cm long, laxly 2–10-flowered; rachis 2–15 cm long, papillate-hairy; floral bracts lanceolate or linear-lanceolate, apex acuminate. Flowers yellowish-green; ovary and pedicel strongly arcuate, 1.5–2 cm long, pubescent. Dorsal sepal forming hood with petals, ovate, concave, 7–9 mm long, 4–5 mm wide, apex subobtuse; lateral sepals reflexed, obliquely ovate, 8–11 mm long, 4–5 mm wide, apex acuminate. Petals shallowly 2-lobed; upper lobe oblique, oblong-lanceolate to ovate-lanceolate, 8–10 mm long, 3–4 mm wide, margin papillate-ciliate, apex subacute; lower lobe ca. 1.5 mm long; lip deeply 3-lobed, spurred, lobes reflexed over base; lateral lobes linear-filiform, 2–4 cm long, curled at apex; mid-lobe linear, 1–2 cm long; spur cylindrical-clavate, 1.5–2.5 cm long, often horizontal and curved upwards. Column stout, anthers parallel; pollinia granular; caudicles stout, elongated; stigma processes lingulate. (Fig. 19).

###### Phenology.

Flowering from June to August.

###### Habitat.

Thickets, alpine grasslands; 3200–4900 m elev.

###### Distribution.

**Figure 20.**
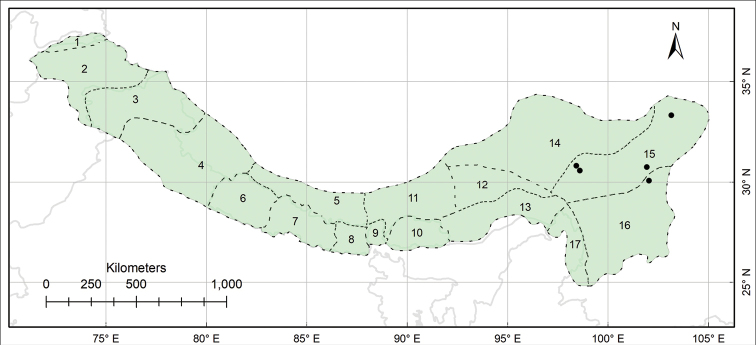
Distribution of *Habenaria tibetica* (black circles) in the Pan-Himalaya.

N and S Hengduan, also in NE Qinghai of China. (Fig. 20).

###### More illustrations.

Wu et al. (2010, fig. 195, 5–7).

###### Additional specimens examined.

**N HENGDUAN**: **Gonjo**, 3200 m elev., *Vegetation Team of Qinghai-Tibet Plateau 9671* (PE); **Gonjo**, 3800 m elev., 2010, *Kham Expedition 10-1872* (PE); **Songpan**, 3835 m elev., 2002, *Y.B. Luo 827* (PE); **Xiaojin**, Hanniu Region, 3400 m elev., 1959, *Z.G. Liu 0359* (PE). **S HENGDUAN**: **Kangding**, Muya Region, A-Tai Xiang, 3560 m elev., 1982, *Lang et al. 981* (PE, KUN).

###### Note.

According to the protologue, *Habenaria tibetica* was described by Schlechter, based on two specimens from China: East Tibet, Ta tsien lu, 3600 m elev., *Limpricht 2303* and Batang-Litang, 4800–4900 m elev., *Limpricht 2277* (Limpricht 1922); consequently, both of these are syntypes as per Art. 9.6 of the ICN (Turland et al. 2018). Many of the type specimens, described by Schlechter together with *H. tibetica* (e.g. *Platanthera minax* Schltr. & *P. winkleriana* Schltr.), were believed to be kept at B and later, their duplicate specimens were recovered at other European herbaria WU and WRSL; however, despite an extensive search, none of the type materials of *H. tibetica* could be located in any of the world’s major herbaria and could have been destroyed during the Second World War at B. Furthermore, we were unable to find any other original material related to the species. Thus, assuming that all the original material of *H. tibetica* is lost, it warrants designating a neotype, which is here accomplished. For that purpose, *X.L. Jiang 36260* (PE) is designated the neotype according to Art. 9.8 of the ICN (Turland et al. 2018); this specimen was also collected from the original type locality (Kangding, Sichuan). The designated neotype specimen conforms to the description in the protologue and is consistent with the current application of the taxon name (e.g. Chen and Cribb 2009).

## Supplementary Material

XML Treatment for Habenaria josephi

XML Treatment for Habenaria diplonema

XML Treatment for Habenaria fargesii

XML Treatment for Habenaria glaucifolia

XML Treatment for Habenaria aitchisonii

XML Treatment for Habenaria balfouriana

XML Treatment for Habenaria szechuanica

XML Treatment for Habenaria tibetica
